# How are medical students using the Electronic Health Record (EHR)?: An analysis of EHR use on an inpatient medicine rotation

**DOI:** 10.1371/journal.pone.0221300

**Published:** 2019-08-16

**Authors:** Jeffrey Chi, Jason Bentley, John Kugler, Jonathan H. Chen

**Affiliations:** 1 Department of Internal Medicine, Division of Hospital Medicine, Stanford University School of Medicine, Stanford, California, United States of America; 2 Quantitative Sciences Unit, Stanford University School of Medicine, Stanford, California, United States of America; University of Alabama at Birmingham, UNITED STATES

## Abstract

Physicians currently spend as much as half of their day in front of the computer. The Electronic Health Record (EHR) has been associated with declining bedside skills and physician burnout. Medical student EHR use has not been well studied or characterized. However, student responsibilities for EHR documentation will likely increase as the Centers for Medicare and Medicaid Services (CMS) most recent provisions now allow student notes for billing which will likely increase the role of medical student use of the EHR over time. To gain a better understanding of how medical students use the EHR at our institution, we retrospectively analyzed 6,692,994 EHR interactions from 49 third-year clerkship medical students and their supervising physicians assigned to the inpatient medicine ward rotation between June 25 2015 and June 24 2016 at a tertiary academic medical center. Medical students spent 4.42 hours (37%) of each day at the on the EHR and 35 minutes logging in from home. Improved understanding of student EHR-use and the effects on well-being warrants further attention, especially as EHR use increases with early trainees.

## Introduction

Physicians today are spending an increasing amount of time at the computer. Studies consistently show that approximately half of each working day is spent in front of a screen, often followed by additional time working from home [1–3]. Electronic Health Record (EHR) use has been implicated in playing a role in physician burnout [4], and associated with the decline of bedside medicine. In some ways, the EHR has become a symbol for many of the frustrations of modern day healthcare.

While nearly all medical schools allow EHR access as part of medical training [5], little is known about how medical students use the EHR during their training. Students may be more prone to spend time in front of the computer when rotating on an internal medicine rotation compared with other specialties [6]. Beyond this, student EHR instruction and use during clinical training has not been well described and can be inconsistent, as acquisition of skills are often self-taught or learned from near-peers. This has prompted calls from the American Medical Association and others, for more oversight in how medical students are taught to use the EHR, especially as it relates to documentation [7, 8].

Residents and attendings have historically been required to chart independently from students until this year with the introduction of CR10412, a new provision from the Centers for Medicare and Medicaid Services (CMS) allowing student notes in their entirety to also be used for billing [9]. With these recent guideline revisions, student EHR use continues to evolve and is likely to only increase over time.

In this study, we aimed to characterize how medical students interact with the EHR during early clinical training by quantifying and describing their EHR use during their inpatient internal medicine rotation. Better understanding of medical student EHR use could help medical educators to better prepare medical students for their interactions with the EHR and optimize learning opportunities while avoiding potential pitfalls.

## Methods

Our institution uses the EPIC EHR system (Madison, WI) which logs provider actions as clinicians navigate through various parts of the medical record. These include, for example, reviewing charts, placing orders, accessing laboratory results, and generating notes. Historical data for the period June 25, 2015 to June 24, 2016 was extracted using our institutional informatics platform [10] and linked with scheduling databases. This approach has been used and validated in prior studies correlating EHR audit logs with time-motion studies [1, 11]

We analyzed EHR interactions for all 49 clerkship medical students that rotated at our teaching hospital during this period. For comparison, data for 34 interns, 16 supervising residents, and 13 hospitalists assigned to the inpatient medicine ward rotation was also analyzed. These providers were assigned to the service more than once throughout the year while students only rotated on service once per year in 4 week blocks. Hospitalists accounted for 65% of attending staffing while twenty sub-specialist attendings with additional outpatient EHR activity were excluded. Provider days in which >30 patient charts were accessed were excluded to reduce the impact of other activity such as quality improvement initiatives and research projects. This resulted in 3.7% (185/4698) of provider days being excluded, providing 6,206,208 EHR interactions across 4783 provider days for analysis.

Hours of activity were calculated by dividing the day into 5-minute intervals and aggregating the intervals with actions logged. Patients assigned to students were identified by the presence of student documentation during the hospitalization. If a patient chart was accessed by a student without documentation, they were labelled as being only peripherally followed. 5-minute intervals were assigned to either the assigned or peripherally followed patient group depending on the majority of actions within that interval, then summed to determine total time spent for each patient group.

All analysis was performed using R, version 3.4.3. This study was reviewed and approved by the Stanford Administrative Panel on Human Subjects in Medical Research.

## Results

Medical student clerkships consist of 4-week periods and follow a clinical schedule that mirrors the resident call schedule where medical teams are “on-call” on days 1 and 4 of a 5 day call cycle. This schedule results, on average through the period, in 4 days off, 11.2 “on-call” 14-hour days, and 12.8 “non-call” days which we conservatively estimated as 10-hour work days. Students are also excused for mandatory teaching conferences and didactics which account for 2–3 hours per day on weekdays. Based on this schedule, we estimate that students worked an average of 11.9 hours per day over the course of the month.

**Fig 1:**
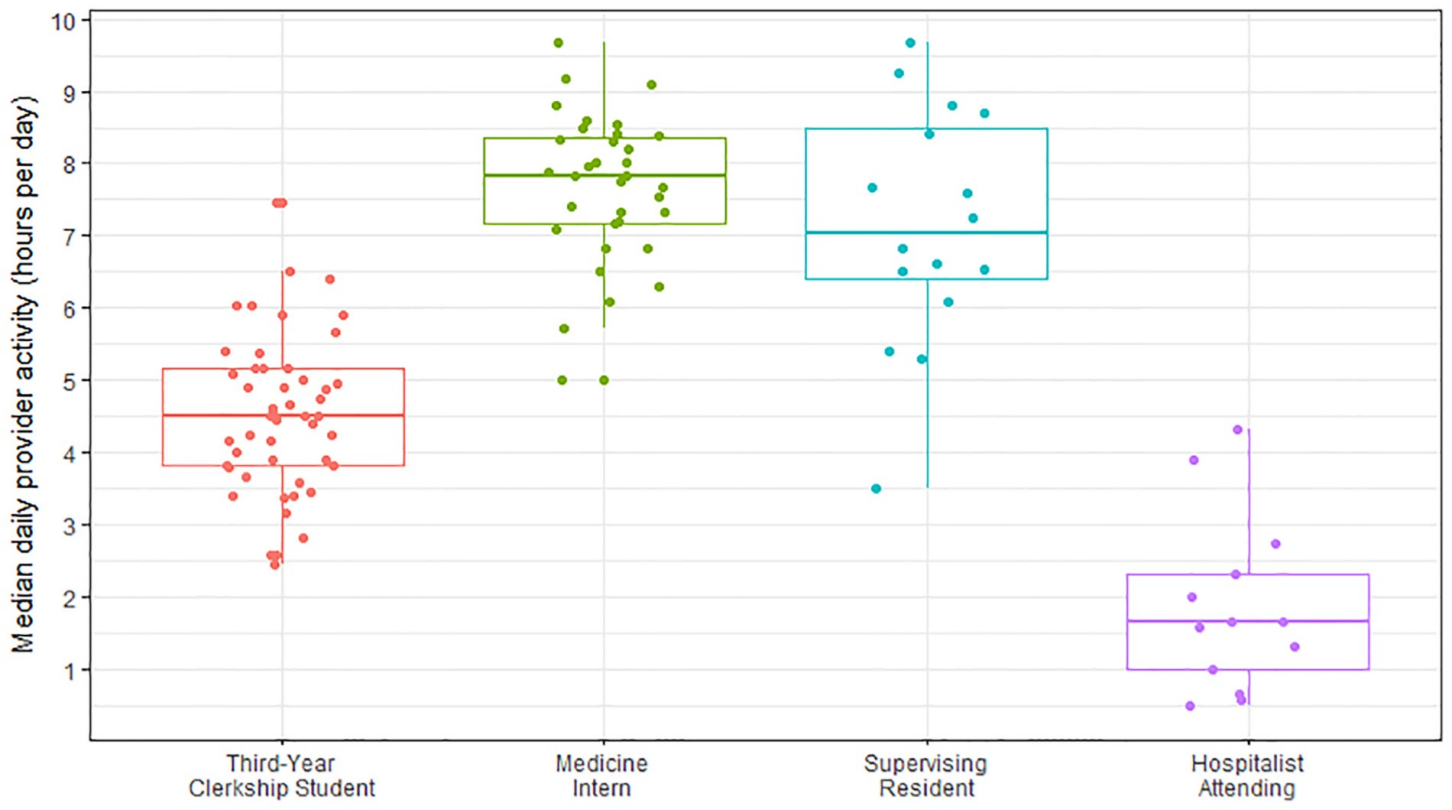
EHR activity by level of training.

Median time spent on the EHR was 4.42 hours for 3^rd^ year clerkship students which was less than interns or residents (p<0.001, Table 1, Fig 1), resulting in a conservative estimate of 37% of time being occupied by EHR use. Students typically wrote a median of 3 (IQR = 2–4) patient notes but accessed 8 (IQR = 4–13) patient charts per day (Table 1). Only 45.1% of student EHR actions were for assigned patients; the remaining activity was for peripherally followed patients. Most student EHR activity focused upon the review of labs/data/reports (44.9%) and notes (20.3%) (Table 1). 89.7% of activity was performed from a team workroom and 7.9% through remote access. 83% of students used a portable workstation or laptop during their rotation but this only accounted for 1.4% of EHR activity, while mobile devices only accounted for 1%.

**Table 1:** Characteristics and daily activity measures summary by provider role.

Characteristics/daily activity measures	Third-Year Clerkship Student	Medicine Intern	Supervising Resident	Hospitalist Attending
**Clinician**	49	34	16	13
**Included scheduled provider days with any action**^a^**, n**	1151	2014	437	1181
**Active days per provider, mean**	23.5 (SD = 2.5)	59.2 (SD = 16.8)	27.3 (SD = 15.4)	90.8 (SD = 44.8)
**Hours of EHR activity per provider day, median (IQR)**	4.42 (3.00–6.17)	7.67 (4.83–9.67)	6.92 (4.08–8.75)	2.08 (1.00–3.33)
**Patient charts accessed per provider day, median (IQR)**	8 (4–13)	10 (7–14)	17 (13–22)	16 (12–21)
**Patients charts documented per provider day, median (IQR)**	3 (2–4)	7 (5–8)	10 (7–13)	10 (5–14)
**Total actions, n**	775487	3492224	758348	1180149
**Actions per provider day, median (IQR)**	615 (384–907)	1817 (1085–2418)	1630 (904–2406)	701 (365–1422)
**Action type, n (column %)**				
Reviewing Labs/Data/Reports	348430 (44.9)	1489944 (42.7)	350388 (46.2)	572001 (48.5)
Writing Notes	88390 (11.4)	192675 (5.5)	19971 (2.6)	56725 (4.8)
Reviewing Notes	157332 (20.3)	475190 (13.6)	115442 (15.2)	186588 (15.8)
Reviewing Orders	36321 (4.7)	127130 (3.6)	21066 (2.8)	16538 (1.4)
Placing Orders	0 (0)	135424 (3.9)	23758 (3.1)	2522 (0.2)
Other	145014 (18.7)	1071861 (30.7)	227723 (30.0)	345775 (29.3)

^a.^ This is the number of days within any actions among scheduled days during the period 25 June 2015 to 24 June 2016 and after excluding any such days with actions logged for more than 30 different patients. SD = Standard Deviation, IQR = Inter-quartile range.

## Discussion

During the 2015–2016 academic year, third-year clerkship students rotating on the internal medicine service at our academic medical center spent 4.42 hours per day using the EHR. Their EHR use more closely resembled that of near-peer housestaff than the attendings. Electronic audits allowed us to study a larger number of providers simultaneously caring for common patients across multiple training levels for a longer duration than prior inpatient observational studies. This also allowed us to account for home computer access and minimize Hawthorne effect bias. However, out study was limited to a single site and findings may differ from other institutions with differences in ward team structures and schedules. Because our electronic audits only captured EHR-related activity, our study may also underestimate overall time spent at the computer such as online searches and other access of electronic resources.

Although EHRs were primarily designed for billing and documentation, our findings suggest that students may also use the EHR for other purposes. Students actively followed and wrote notes for 3 patients per day, but additional patients on the team were peripherally followed which accounted for almost half of their total EHR activity. The EHR could thus be serving as an educational resource as tracking additional patients can expand the volume and variety of cases students are exposed to and add to learning beyond the immediate patient encounter [12]. Documentation from experienced physicians and consultants can serve as a resource for students while access to real-time data can empower students to feel more involved as they are often not the primary contact for patient care. However, these findings also support anecdotal observations that students may indeed, feel more comfortable learning from patients virtually rather than seek out learning opportunities at the bedside [13].

It may be surprising to some to see that medical students, still protected from many burdens of indirect care, still spent substantial time at the computer. Educators have also long been concerned with this growing trend [14] as there are unfortunately, often evaluation-based incentives to prioritize the chart over the patient. Students are often judged on their capacity to “function like interns” and may be implicitly encouraged to role-model resident behavior. For the struggling student, the chart can serve as a crutch and mask knowledge gaps; knowing a patient’s detailed medical record often passes for knowing the patient themselves [13]

Aside from presentations on rounds, student clerkship performance is often evaluated based on the quality of documentation. It thus surprising to find that only 11.4% of student EHR activity revolved around note-writing. Interestingly, prior research has shown that most EHR notes today are not written *de-novo* and are often computer-generated with little original content [15]. While our study did not specifically assess for the usage of templates and copy/paste, student documentation activity was actually similar to that of interns when corrected for number of patients which could imply similar documentation practices. This may warrant further attention, given that over-reliance on these EHR tools by less experienced trainees can deprive students of valuable learning opportunities derived from note-writing. Faculty also face additional challenges in student assessment and providing meaningful feedback when they are not able to differentiate their notes from others. Additionally, students did not receive much practice with order entry, which might also impact their preparation for future responsibilities [16].

The EHR has changed how medicine is practiced and student experiences with the EHR early-on are likely to have a long-term impact. Medical students at our institution spent 4.42 (3.00–6.17) hours per day interacting with the EHR, primarily focused upon chart review rather than documentation or order entry. Without guidance, the EHR can lead to unintended consequences [17, 18] and students would likely benefit from consistent observation and a structured curriculum for proper use. Further understanding of how the EHR can help and hinder learners may need to begin even earlier in training than previously believed in order to prepare for future practice.

## Supporting information

S1 File This file contains the deidentified EHR usage data by providers during the study period. (XLSX)
